# HER2 Overexpression in Periampullary Tumors According to Anatomical and Histological Classification—A Systematic Review

**DOI:** 10.3390/jpm14050463

**Published:** 2024-04-27

**Authors:** Ioan Cătălin Bodea, Andra Ciocan, Florin Vasile Zaharie, Raluca Bodea, Florin Graur, Ștefan Ursu, Răzvan Alexandru Ciocan, Nadim Al Hajjar

**Affiliations:** 1 Department of Surgery, “Iuliu Hațieganu” University of Medicine and Pharmacy, Croitorilor Street, No. 19–21, 400162 Cluj-Napoca, Romania; bodea_ioan_catalin@elearn.umfcluj.ro (I.C.B.); zaharie.vasile@umfcluj.ro (F.V.Z.); florin.graur@umfcluj.ro (F.G.); ursu_stefan@elearn.umfcluj.ro (Ș.U.); nadim.alhajjar@umfcluj.ro (N.A.H.); 2 “Octavian Fodor” Regional Institute of Gastroenterology and Hepatology, Croitorilor Street, No. 19–21, 400162 Cluj-Napoca, Romania; ralucabodea@yahoo.com; 3 Department of Surgery-Practical Abilities, “Iuliu Hațieganu” University of Medicine and Pharmacy, Marinescu Street, No. 23, 400337 Cluj-Napoca, Romania; razvan.ciocan@umfcluj.ro

**Keywords:** pancreatic cancer, protein receptors, HER2, overexpression, molecular pathway

## Abstract

Pancreatic cancer is one of the most aggressive, heterogeneous, and fatal types of human cancer; therefore, more effective therapeutic drugs are urgently needed. Human epidermal growth factor receptor 2 (HER2) overexpression and amplification have been identified as a cornerstone in this pathology. The aim of this review is to identify HER2 membrane overexpression in relation to pancreatic cancer pathways that can be used in order to develop a targeted therapy. After searching the keywords, 174 articles were found during a time span of 10 years, between 2013 and 2023, but only twelve scientific papers were qualified for this investigation. The new era of biomolecular research found a significant relationship between HER2 overexpression and pancreatic cancer cells in 25–30% of cases. The variables are dependent on tumor-derived cells, with differences in receptor overexpression between PDAC (pancreatic ductal adenocarcinoma), BTC (biliary tract cancer), ampullary carcinoma, and PNETs (pancreatic neuroendocrine tumors). HER2 overexpression is frequently encountered in human pancreatic carcinoma cell lines, and the ERBB family is one of the targets in the near future of therapy, with good results in phase I, II, and III studies evaluating downregulation and tumor downstaging, respectively.

## 1. Introduction

Pancreatic cancer (PC) is the seventh leading cause of cancer-related deaths worldwide, comprising 2.5% of all forms of cancer [1]. However, the heterogeneity of the disease makes the incidence higher in developed countries. The reasons for the vast differences in mortality rates of periampullary tumors are not yet completely understood, but they may be due to a lack of appropriate diagnosis, treatment, and follow-up [1]. Periampullary tumors are a widely used term to define a heterogeneous group of neoplasms deriving from completely different types of primordial cells: head of the pancreas (PDAC/PNET), distal common bile duct (BTC), and ampulla of Vater (ampullary carcinoma). This term should be correctly distinguished from ampullary carcinoma, or ampulloma, which is a tumor topographically centered in the region of the ampulla of Vater, deriving from the duodenal mucosal cells. Despite accounting for less than 10% of the total length of the small intestine, the duodenum, especially D2 (the second portion of it), is the site of 25% to 45% of all small bowel cancers. Ampullary carcinoma is a relatively uncommon neoplasm that accounts for approximately 6% of the total periampullary malignancies, with an incidence estimated at 2.9 cases per million in the general population and accounting for approximately 0.2% of gastrointestinal tract carcinomas. Distal cholangiocarcinoma is a rare malignancy of the biliary tract, occurring in 27% of total extrahepatic biliary adenocarcinomas [1,2]. With a 5-year survival rate of only 9% for pancreatic cancer and even lower depending on the histopathological type [2], this neoplasia caused 432, 242 new deaths in 2018 alone, and 458,918 new cases of pancreatic cancer are diagnosed every year [2,3]. To date, the etiology of pancreatic carcinoma is still insufficiently described due to the multitude of incriminating factors, although certain risk factors have been identified, such as diabetes mellitus, tobacco, dietary elements, alcohol abuse, obesity, age, ethnicity, family history, genetic factors, Helicobacter pylori infection, non-0 blood group, and chronic pancreatitis [4]. Surgery remains the main curative treatment, but only 15–20% of tumors are resectable at the time of diagnosis, and the prognosis is still poor, even for R0 resections [5,6,7]. Adjuvant chemotherapy seems to provide moderate improvements, with a medium survival of about 12 months; the regimen has not yet established favorable results [8,9]. Therefore, it is imperative to provide targeted therapy in order to improve overall and disease-free survival. Multiple studies are searching for the key components of protein receptors in the abnormal proliferation of neoplastic cells. Growth factors are essential for the development, growth, and homeostasis of multicellular organisms. Acting through cell surface receptors, growth factors are required for cell-to-cell communication, sustaining embryonic tissue induction, cell survival, apoptosis, tissue specialization, and cell migration. The epidermal growth factor family consists of four members: EGFR (ErbB1, HER1), ErbB2 (HER2, neu in rodents), ErbB3 (HER3), and ErbB4 (HER4). ErbB2, however, is unique in that it has no known ligand but is the preferred dimerization partner for other EGFRs [6,7]. *HER2* is a protooncogene located on chromosome 17 at q21 that encodes epidermal growth factor receptors with tyrosine kinase activity. In breast cancer, the *HER2* gene is amplified in 15%–20% of invasive breast cancers, and its amplification is closely linked to HER2 protein overexpression [10]. *HER2* amplification is a poor prognostic factor associated with a high rate of recurrence and mortality and is a predictive factor for response to certain chemotherapies [1,2]. Most importantly, it is a sole predictive marker for treatment benefits from HER2-targeting agents such as trastuzumab, lapatinib, and pertuzumab. As HER2-targeted therapy is exclusively effective in HER2-overexpressed and/or HER2-amplified breast cancers, precise assessment of HER2 status is an essential step in breast cancer treatment. The role of *HER2* in digestive tumors regarding both prognostic and therapeutic purposes provides heterogeneous results [11,12]. Novel testing and treatment guidelines are introducing HER2 evaluation in gastric and colorectal cancer, but for periampullary tumors, this protein receptor is less investigated. The rate of HER2 positivity in BTC (biliary tract cancers) has been reported to be 5–20% and between 5–50% for PDAC (pancreatic ductal adenocarcinoma). Case reports and clinical trials declare a good response to HER2 inhibitors as a treatment strategy, with abnormal cell proliferation reduction and tumor downstaging [10,13,14,15]. Above all carcinogenic pathways, the *HER2* pathway seems to be one of the most underestimated and undiagnosed protein receptors in pancreatic cancer. The main scope of this study is to bring together all the available information regarding HER2 overexpression in relation to tumor type, pancreatic ductal adenocarcinoma, distal cholangiocarcinoma, and ampullary tumor. Its overexpression and gene amplification are involved in tumor growth by cell proliferation and cell differentiation from normal into neoplastic ones [16,17,18,19,20]. HER2 lacks specific ligands, so it can form heterodimers only when activated by another receptor, such as EGFR, HER3, and HER4. In addition to abnormal overexpression, this receptor is able to spontaneously homodimerize, compared to HER3 or HER4 [21,22]. Worldwide, research attempts exist to identify the exact role of this receptor in abnormal cell proliferation in pancreatic cancer and periampullary tumors in general for anti-HER2 targeted therapy development. There is no standard therapy at the moment for HER2-positive pancreatic cancer patients, and this is the reason for our focus.

The aim of this review is to identify the overexpression of the HER2 protein receptor in relation to cephalopancreatic cancer and periampullary tumors in order to find out its role in cell proliferation. Consequently, the systematic review follows two trends of research—the role of the *HER2* pathway in pancreatic carcinogenesis and the rate of HER2 positivity according to the following tumor subtypes: pancreatic adenocarcinoma, distal cholangiocarcinoma, and ampullary carcinoma.

## 2. Materials and Methods

**Figure 1:**
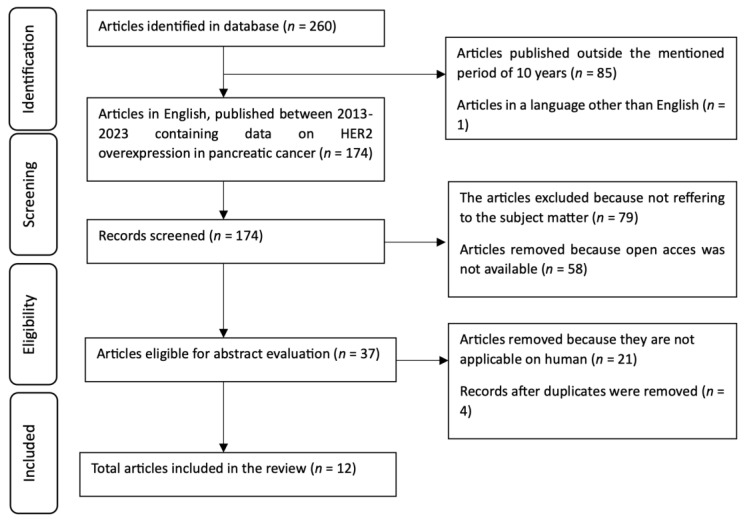
PRISMA flow diagram for the selected studies included in the systematic review identified in three databases, i.e., PubMed, Embase, and Clarivate Web of Science.

The methodology of this systematic review consists of defining search algorithms, selection criteria, and data extraction protocols. The present research followed the guidelines outlined in the Preferred Reporting Items for Systematic Reviews and Meta-Analyses (PRISMA) statement (Figure 1). From January 2013 until December 2023, articles published in English in the online databases PubMed (Medline), Embase, and Clarivate Web of Science were analyzed. This systematic review has not been registered.

The research was performed using the following keywords: “pancreatic cancer” AND “protein receptors” AND “HER2” using “AND” and “OR” between the mentioned elements as Boolean operators. All titles referred to in English and published over a precise period of 10 years were assessed for eligibility by title and abstract by three separate researchers to reduce bias.

The inclusion criteria follow studies containing information about HER2 overexpression, the HER2 pathway, protein receptors in pancreatic cancer, distal cholangiocarcinoma, ampullary carcinoma, and pancreatic carcinogenesis. All studies were conducted on human cell lines. Exclusion criteria meant articles published earlier in 2013, not referring to the subject, letters to the editor, short reports, narrative reviews, non-English papers, articles focusing on *HER2* in other types of cancer (such as breast cancer, gastric cancer, and colorectal cancer), studies about other pathways in pancreatic cancer, and studies focusing on therapeutic effects on different cell lines and cell proliferation. For the risk of bias evaluation, we applied the updated quality assessment for diagnostic accuracy studies-2 (QUADAS-2) method to each article selected for analysis. It covered five areas of bias: patient selection, index test, reference standard, subject flow, and timing. Microsoft Excel’s extraction tool was used for data collection and database settlement. For the quality evaluation, we utilized standardized criteria, including study participation, factor measurement, value, and applicability. In the process of data selection and extraction, any inconsistencies identified by the three primary reviewers were subjected to further examination by two additional ones. The list of references for specific research projects was systematically examined to identify potential publications, following the snowball technique.

Using the above-mentioned keywords for search, 260 articles were found. By reducing the timeframe to 10 years, from 2013 until 2023, a number of 175 articles containing data on pancreatic cancer, cholangiocarcinoma, ampullary carcinoma, receptors, and HER2 were found. From this selection of articles, 1 article was removed because it was not written in English, and 79 were removed because they were not referring to the subject matter. Furthermore, 21 studies were excluded because they did not involve human subjects. A total of 58 articles were discarded because they were not open access. Moreover, 4 articles were removed because they were duplicates between the databases; therefore, 12 scientific papers remained eligible for the systematic review.

This systematic review has several limitations, mainly linked to inter-study heterogeneity. In some articles, a clear definition of the primary tumor site was not available, or the results were not reported separately for each subgroup, thus limiting the eligible data for inclusion in subgroup analysis. Since there are no standardized techniques and scores to assess HER2 amplification and expression available in PC and because there are no internationally established and validated methods for HER2 testing in any type of tumor, inconsistency in methodology may be an important issue. Differences in methodology, disease stage (early vs. advanced), tumor specimen (resection specimen vs. biopsy), site of tumor specimen (primary vs. metastatic), and IHC and ISH technical issues may explain the wide range of HER2 expression rate (1.1% to 76%) reported in this review. Finally, no survival data were available, making it impossible to assess the prognostic implications of HER2 overexpression or amplification.

## 3. Results

All articles included in the study (n = 12) focused on identifying the overexpression of the HER2 protein receptor in different cancer cell lines in relation to the histopathological type and possible upcoming targeted therapies. Among the evaluated articles, there were different HER2 determinations applied to inhomogeneous cohorts. All articles used human tissue or human cell lines obtained either by EUS (endoscopic ultrasound) biopsy—FNAC (fine needle aspiration cytology)—or resected samples after pancreatoduodenectomies. Protein receptor pathways involved in periampullary tumor development were analyzed, including the entire epidermal growth factor family, with a focus on HER2 overexpression and amplification, in order to provide quality data regarding targeted therapy against these dreadful tumors.

Each article examined a certain member of the growth family, like EGF, EGFR, HER1, HER2, HER3, and HER4, in accordance with a certain tumoral subtype; their aim is shown in Table 1. 

Akihiro et al. [33] have demonstrated an overexpression rate of 10–20% for HER2 in biliary tree carcinomas, making trastuzumab–deruxtecan more active than FOLFIRINOX in difficult-to-treat, unresponsive, and HER2-expressing cases (Table 2). In addition, Majid et al. [28] demonstrated that dacomitinib, an irreversible tyrosine kinase inhibitor, exhibits stronger anti-tumor efficacy than single-targeted ERBB inhibitors in PDAC. Tanzel et al. [30] identified that afatinib in combination with the insulin-like growth factor 1 receptor (IGF-IR) inhibitor NVP-AEW541 resulted in a synergistic growth inhibition of pancreatic cancer cells. Assenat et al. [29] show that combining gemcitabine, trastuzumab, and erlotinib is more effective in terms of disease control rate, progression-free survival, and overall survival. The present systematic review reveals that sole inhibition of HER2 may not be enough because of the consequent upregulation of other ERBB family members, which can stimulate abnormal cell proliferation. In their study, Elebro et al. [26] demonstrated that the HER2-HER3 homodimer is a powerful activator of the PI3K/Akt pathway, causing aberrant proliferative and antiapoptotic intracellular signals. Inhibition of HER2 activity, however, causes upregulation of HER3, so simultaneous blockage of HER2 and HER3 activity gives more potent tumor cell inhibition than one receptor blockage alone. 

Regarding the pathways targeted and drug agents used in their studies, authors favored topoisomerase inhibitors, panERBB receptor inhibitors and tyrosine kinase inhibitors to reduce cancer cell proliferation and increase survival (Table 3). The most frequently used inhibitory agent was trastuzumab. Tanzeel et al. [30] mentioned that the combination of dinaciclib, afatinib, and an insulin-like growth factor 1 receptor inhibitor is the most effective downstaging treatment in pancreatic cancer. Majid et al. [28] show that dacomitinib induces cell apoptosis in PDAC by inhibiting XIAP, cIAP1, and cIAP2. 

A large variation in the number of patients included in the cohorts evaluated in each study exists. Furthermore, there is no specific histopathological division between distal cholangiocarcinoma and suprapancreatic cholangiocarcinoma regarding BTC.

In pancreatic cancer, Rabia et al. [31] showed that simultaneous targeting of EGFR and *HER2* with cetuximab and trastuzumab induces a synergistic therapeutic effect. They extended their strategy by adding pertuzumab (an anti-HER2 antibody) to 9F7-F11 (an anti-HER3 antibody) in PDAC. The monoclonal antibody combination blocked MAPK and AKT pathways by disrupting hetero- and homodimers and induced receptor down-regulation.

Regarding the technique used for HER2 detection, 8 out of 12 authors used immunohistochemistry, i.e., Tanzeel et al. [30] used flow cytometry; Marinovic et al. [32] used SPN genotyping; Majid et al. [28] used MTT assay-cell metabolic activity; and Elebro et al. [26] used tissue microarrays (TMAs). Moreover, Akihiro, Nicola, and Salvatore used both IHC and ISH to reveal HER2 overexpression. The researchers struggle to find the best technique in terms of both qualitative and quantitative values, according to purpose and funds [24,27,33].

HER2 positivity rates vary largely from study to study, mainly due to the differences in sample quality and cohort size. Akihiro et al. [33] included 32 patients in their study, compared to Salvatore et al. [27], who conducted the research on 920 patients (Table 2). Consequently, HER2 positivity rates oscillate between 1.1 and 76% in PDAC, with an average of 30%, and 5 to 76% in BTC. For pancreatic neuroendocrine tumors, HER+ ranges between 35 and 40%, more than the median of PDAC. Marinovic et al. [32] found a median of 37.9% in nonfunctional neuroendocrine tumors and 36.11% in functional tumors (Table 4). Xiaoping et al. [25] discovered that in some samples there is not just one ERBB receptor of the family upregulated or overexpressed but two or three receptors, which co-stimulate abnormal cell proliferation. The authors determined HER2+ in 24% of PDAC cases and the combination of EGFR, HER2 2+, and HER3 2+ in 11%. 

Akihiro et al. showed that trastuzumab deruxtecan is more effective than standard chemotherapy (FOLFOX) in difficult-to-treat HER2+ cases of cholangiocarcinoma. In particular, trastuzumab has greatly improved the therapeutic approach of advanced BTC but has little evidence in other digestive cancers, as proved by Nicola et al. Trastuzumab in combination with cytotoxic therapies in HER2-positive BTC has increased the tumor response rate from 35% to 47%, improving progression-free survival from 5.5 months to 6.7 months and overall survival from 11.1 months to 13.8 months. The combination of pertuzumab and trastuzumab has been reported to induce a synergistic inhibition of in vivo tumor growth in BTC due to a more complex blockade of HER2/HER3 signaling, as shown in Salvatore et al. All chemo- and immunotherapics are thoroughly explained in File S1 from the Supplementary Materials.

Comparing trastuzumab plus chemotherapy versus chemotherapy alone, de Vries et al. showed an overall survival benefit with this combination instead of chemotherapy alone, regardless of variability in HER2 staining, with a numerically greater benefit in patients with less variable HER2 protein expression (>30% of stained cells), out of which only one patient with PDAC and two with BTC had strong HER2+ and 3+ tumors.

Assenat et al. proved that increased co-expression of EGFR and HER2 is associated with advanced tumor stage, aggressive phenotype, the presence of distant metastases, and shorter overall survival. Therefore, the combination of gemcitabine, trastuzumab, and erlotinib is more effective in terms of disease control rate, progression-free survival, and overall survival. The triplet has not reported unexpected toxicity results, except for a higher rate of thromboembolic complications (33%) than usually described in metastatic PDAC (15–20%), which is similar to the combination of gemcitabine and nab-paclitaxel.

In their study, Rabia et al. described that tumor resistance to gemcitabine is accompanied by HER2 and HER3 overexpression. Simultaneous targeting of EGFR and HER2 with cetuximab and trastuzumab induces a synergistic therapeutic effect. Afatinib is more effective than erlotinib in inhibiting growth factors in PDAC, more so in combination with the insulin-like growth factor 1 receptor (IGF-IR).

## 4. Discussion

Despite major advances in the early diagnosis of solid tumors in the past three decades, pancreatic cancer and distal cholangiocarcinoma, likewise, remain challenging in terms of curative treatment [35]. By 2030, it is predicted to become the second-leading cause of cancer-related deaths after lung cancer [36]. Due to their heterogeneous nature, retroperitoneal location, non-specific symptoms, and lack of screening methods, the overwhelming majority of cephalopancreatic tumors are diagnosed in advanced stages [37]. Until now, only a few HER inhibitors and EGFR-specific tyrosine kinase inhibitors (TKI), like erlotinib, lapatinib, and gefitinib, have gained FDA approval for the targeted therapy of locally advanced, unresectable, or metastatic pancreatic cancer in combination with the cytotoxic agent gemcitabine. Erlotinib and gefitinib are most effective on EGFR, and lapatinib is equally active against EGFR and HER2. Because of the late diagnosis and untargeted treatment, most of these patients have a low survival rate of 10% at 5 years from diagnosis. Almost 60–70% of PDAC cases are localized cephalopancreatic. It is mandatory to develop a good algorithm for neoadjuvant treatment for tumor downstaging and downsizing targeted at overexpressed receptor inhibition or downregulation. Similar to HER2+ therapy used in breast and gastric cancer with good results, the biomolecular substrate of pancreatic cancer may include HER2 targeted treatment. Since not only a single receptor is overexpressed in pancreatic cancer, additional information regarding ERBB is needed. This resides in the entire HER family expression analysis to be able to block the whole HER signaling network and increase the anti-tumor response. Most of the drug combinations effectively decrease cell proliferation in experimental animal models, but their clinical efficiency needs to be demonstrated [38,39,40].

The tyrosine kinase function of ERBB members is the key to intracellular signaling, activation, and cell transformation, which can work as target agents for tyrosine kinase inhibitors (TKIs). The binding characteristics of each TKI vary according to a specific EGFR mutation, and the choice of TKI agents is based on the unique molecular features of the tumor. Lapatinib connects to the inactive conformation of EGFR with a particularly tight binding that affords a slow off-rate [41]. In a study conducted by Akihiro et al., trastuzumab deruxtecan is the next TKI to be the standard second line of targeted treatment, proving benefit in HER2-positive unresectable or recurrent BTC. In a phase I study, trastuzumab deruxtecan led to an almost 80% reduction in tumor size in patients with distal cholangiocarcinoma. These data suggest that there may be promise in targeting HER2 in non-breast/non-gastric carcinomas [28,42,43]. Chemo- and immunotherapy combinations provide better results in terms of EGFR-HER2 or HER2-HER3 heterodimer disorganization, more efficient and stable inhibition of signaling pathways, and increased degradation of HER2 receptors [16]. Afatinib, a pan-HER blocker, is more effective than erlotinib in inhibiting pancreatic cancer cell proliferation, as reported by Tanzeel et al. Furthermore, of all agents examined in their study, CDK1/2/5/9 inhibitor–dinaciclib was the most potent and effective agent against both primary and metastatic cell proliferation in all periampullary tumors. Improved survival of normal pancreatic cells, a high apoptosis rate, and enhanced radiosensitivity are shown by Majid et al. by treating the PDAC cells with dacomitinib. Moreover, it demonstrated that the anti-tumor activity of single-targeted ERBBs like cetuximab, trastuzumab, and erlotinib was marginal in downgrading the PDAC microenvironment. These hypotheses suggest that for better results, pan-ERBB inhibitors may yield stronger anti-tumoral activity than a single targeted ERBB agent.

Increased co-expression of EGFR and HER2 is associated with advanced tumor stages, aggressive phenotypes, distant metastases, and shorter overall survival. Furthermore, co-expression of HER3 and HER4 is associated with increased tumor size, advanced tumor stage, and high invasiveness [44,45]. Local tumor progression and early systemic dissemination of PDAC cells contribute to a bad prognosis, which is one of the major hallmarks of the disease. Elebro et al. reported that HER3 expression is a favorable prognostic factor in the ampulla of Vater carcinoma–intestinal type, and EGFR overexpression is an adverse one. In pancreato-biliary type, EGFR was found to be a negative prognostic factor, implying resistance to adjuvant gemcitabine [46]. Moreover, HER2 overexpression is an important prognostic factor both for the intestinal and pancreato-biliary types of periampullary tumors. Taking all this into consideration, their statistical significance is moderate when we consider these associations independent of other prognostic factors.

For pancreatic neuroendocrine tumors (PNETs), Marinovic et al. emphasized that the combination of EGF, EGFR, and HER2 plays a significant role in carcinogenesis and impacts susceptibility to various treatment strategies. EGFR overexpression is associated with aggressive types of gastrinoma-functional PNET, similar to PDAC. However, none of the variant genotypes of EGF, EGFR, or HER2 is associated with distant metastases. When it comes to HER2 overexpression in PNETs, there is equal dissemination between functional and nonfunctional tumors. Furthermore, insulinomas demonstrate approximately 42.31% HER2 positivity, higher than the median range, and they may be considered herapeutic targets in PNET because of their high incidence. Tanzeel et al. reported that CDK inhibitor dinaciclib, an irreversible pan-HER tyrosine kinase inhibitor afatinib, and SRC tyrosine kinase inhibitor dasatinib were the most effective agents, inhibiting cell proliferation and migration.

Because there are a multitude of pathological pathways and protein receptors, blocking just one pathway is not effective because carcinogenesis consists of a cumulation of biomolecular alterations; therefore, the use of agents in combination is the key.

For BTC, there is no specific difference between distal cholangiocarcinoma and main biliary duct tumors of the suprapancreatic area. There is no variation in protein receptors between the ampulla of Vater carcinomas and duodenal tumors invading the head of the pancreas. Salvatore et al. described a 17.4% positivity rate of HER2 overexpression in BTC and 27.9% in ampulla Vater carcinoma, in line with the median range of HER2+ in PDAC.

Regarding the method used for HER2 overexpression assay and analysis, IHC (immunohistochemistry) and ISH (in situ hybridization) are frequently used for formalin-fixed paraffin-embedded tissue. IHC measures the total number of HER2 receptors on the cell’s surface for receptor overexpression quantification, and ISH detects the number of copies of the *HER2* gene evidenced in tumor cells’ nuclei. Currently, there is no standard guideline for HER2 status evaluation in pancreatic cancer, hence the necessity to develop a standard procedure in order to efficiently compare results. Because of the heterogeneity of the cohorts and the lack of a standard HER2 assay method, it is hard to compare the results of multiple studies because they use different diagnostic methods.

Nagarkar et al. studied and presented a case report of HER2-positive distal common bile duct carcinoma with multiple relapses and overexpression of the *HER2* gene, which was used as a potential target to treat the patient with trastuzumab. The combination of trastuzumab with cyclophosphamide and methotrexate yielded an excellent treatment response, with the patient remaining in complete response until the last follow-up [47].

Before considering chemotherapy, a complete proteomic profile of overexpressed surface receptors in the cell membrane is necessary. For that, a preoperative biopsy is performed to analyze HER2 overexpression. Studying the effects of combining pan-HER with standard-of-care agents, such as gemcitabine or FOLFIRINOX, is essential. Specific targeted therapy is also needed to downregulate and decrease tumor burden in order to restore it to surgical resectability.

The limitation of the study resides in the fact that various articles provide heterogeneous results. They all convey that HER2 overexpression plays an important role in tumor growth, cell invasion, and proliferation, but with different statistical data. Elebro et al. show 58.3% of HER2+ in 62 patients, compared to a minimum rate of 2.1% found by Xiaoping et al. These major discrepancies come from the distinct cohorts in various articles, the lack of standard diagnostic protocols, and the HER2 overexpression analysis method, making the results difficult to compare between studies. Moreover, the sample size and quality might generate inconsistencies in IHC or ISH positivity.

## 5. Conclusions

Pancreatic cancer remains one of the deadliest cancer types worldwide. During carcinogenesis, HER2 has been identified as an oncogene involved in the upregulation of tumor cell proliferation and invasion. HER2 overexpression has been found in 20–30% of all periampullary tumors, highly dependent upon the histopathological type. Inhibiting the pan-ERBB receptors increases disease control and reduces PDAC growth, with a favorable prognosis. Combined chemotherapy and immunotherapy in HER2+ tumors is a new approach involving simultaneous targeting of each receptor pathway of the HER family. Therefore, HER2 overexpression should be routinely evaluated in cephalopancreatic adenocarcinomas, distal cholangiocarcinomas, and ampullary carcinomas prior to surgery or chemotherapy in order to have a correct indication for systemic treatment and provide targeted treatment.

## Figures and Tables

**Table 1:** Author, year of publication, title of the article, and aim of the study.

Authors	Year of Publication	Title	Aim of the Study
Bittoni et al. [23]	2015	HER family Receptor Expression and Prognosis in Pancreatic Cancer	Identifying HER family overexpression in PDAC
Nicola et al. [24]	2016	HER2 aberrations and heterogeneity in cancers of the digestive system: Implications for pathologists and gastroenterologists	Identifying HER2 aberrations and overexpression in periampullary tumors
Xiaoping et al. [25]	2016	Prognostic role of HER2 amplification based on fluorescence in situ hybridization (FISH) in pancreatic ductal adenocarcinoma (PDAC): a meta-analysis	Identifying the prognostic role of HER2 positivity in PDAC
Elebro et al. [26]	2016	Expression and Prognostic Significance of Human Epidermal Growth Factor Receptors 1, 2 and 3 in Periampullary Adenocarcinoma	Identifying EGFR, HER2, and HER3 expression and HER2 amplification status in periampullary tumors
Salvatore et al. [27]	2017	HER2/HER3 pathway in biliary tract malignancies; systematic review and meta-analysis: a potential therapeutic target?	Identifying the HER2 overexpression rate in BTC
Majid et al. [28]	2019	The ERBB receptor inhibitor dacomitinib suppresses proliferation and invasion of pancreatic ductal adenocarcinoma cells	Evaluating dacomitinib’s effect upon EGFR, HER2, and HER3 expression in PDAC
Assenat et al. [29]	2020	Phase II study evaluating the association of gemcitabine, trastuzumab and erlotinib as first-line treatment in patients with metastatic pancreatic adenocarcinoma (GATE 1)	Evaluating survival rate in patients with HER2+ advanced PDAC
Tanzeel et al. [30]	2020	Synergistic activity of agents targeting growth factor receptors, CDKs and downstream signaling molecules in a panel of pancreatic cancer cell lines and the identification of antagonistic combinations: Implications for future clinical trials in pancreatic cancer	Identifying cells’ surface expression of various growth factor receptors in PDAC
Rabia et al. [31]	2021	Anti-tumoral activity of the Pan-HER (Sym013) antibody mixture in gemcitabine-resistant pancreatic cancer models	Identifying and understanding the pathways of the pan-HER antibody family in PDAC
Marinovic et al. [32]	2022	Analysis of polymorphisms in EGF, EGFR and *HER2* genes in pancreatic neuroendocrine tumors (PNETs)	Identifying the overexpression of EGF, EGFR, and HER2 receptors in PNET
Akihiro et al. [33]	2022	Multicenter phase II trial of trastuzumab deruxtecan for HER2-positive unresectable or recurrent biliary tract cancer: HERB trial	Identifying the benefit of trastuzumab deruxtecan on the ORR in HER2-positive BTC
de Vries et al. [34]	2023	Phase II study (KAMELEON) of single-agent T-DM1 in patients with HER2-positive advanced urothelial bladder cancer or pancreatic cancer/cholangiocarcinoma	Identifying the relationship between biomarkers and tumor response to treatment in PDAC and BTC

*HER2*, human epidermal growth factor receptor 2; HER3, human epidermal growth factor receptor 3; EGFR, epidermal growth factor receptor; ORR, objective response rate; T-DM1, antibody-drug conjugate trastuzumab emtansine; FISH, fluorescence in situ hybridization; PDAC, pancreatic ductal adenocarcinoma; BTC, biliary tract cancer; CDKs, cyclin-dependent kinase inhibitors; PNET, pancreatic neuroendocrine tumors.

**Table 2:** Authors, pathways targeted, drug agents used, number of patients related to carcinoma type, and detection methods used.

Authors	Pathways and Drugs Used	Number of Patients	Detection Method
Bittoni et al. [23]	N.A.	91 PDAC	IHC 1+
Nicola et al. [24]	Topoisomerase inhibition Trastuzumab deruxtecan + Capecitabine	17 PDAC	IHC2+ and 3+ ISH 2+ and 3+
Xiaoping et al. [25]	N.A.	764 PDAC	ISH 1+ and 2+
Elebro et al. [26]	N.A.	175 PDAC + BTC + AVC	(TMAs) IHC 2+ and 3+
Salvatore et al. [27]	N.A.	920 BTC 303 AVC	IHC2+ and 3+ ISH 2+ and 3+
Majid et al. [28]	ERBB inhibition Dacomitinib + lapatinib	96 PDAC	MTT assay
Assenat et al. [29]	Tyrosine kinase inhibition Erlotinib + Trastuzumab + Gemcitabine	62 PDAC	IHC 2+ and 3+
Tanzeel et al. [30]	Tyrosine kinase inhibition Dinaciclib + afatinib + dasatinib	96 PDAC	Flow cytometry (fluorescence intensity MFI)
Rabia et al. [31]	Pan-HER Inhibition	45 PDAC (Gemcitabine-resistant cells)	IHC 2+ and 3+
Marinovic et al. [32]	N.A.	68 PNETs	SNP genotyping
Akihiro et al. [33]	Topoisomerase inhibition Trastuzumab deruxtecan	32 BTC	IHC 2+ and 3+ ISH 2+ and 3+
de Vries et al. [34]	Topoisomerase inhibition Trastuzumab emtansine	133 PDAC	IHC 3+
		82 BTC	

SNP, single nucleotide polymorphism; TMAs, tissue microarrays; MTT assay, cell metabolic activity; FISH, fluorescence in situ hybridization; IHC, immunohistochemistry; PNETs, pancreatic neuroendocrine tumors; PDAC, pancreatic ductal adenocarcinoma; BTC, biliary tract cancer/cholangiocarcinoma; AVC, ampulla of Vater carcinoma.

**Table 3:** Author and significant therapeutic findings.

Title	Immunotherapy vs. Chemotherapy in *HER2+*
Bittoni et al. [23]	Trastuzumab + Capecitabine > Capecitabine alone in PDAC
Nicola et al. [24]	Trastuzumab-emtansine + Gemcitabine > Gemcitabine alone in BTC and metastatic digestive tumors
Xiaoping et al. [25]	Trastuzumab + gemcitabine > chemotherapy + gemcitabine in PDAC
Elebro et al. [26]	Erlotinib + gemcitabine > chemotherapy + gemcitabine in BTC and PDAC
Salvatore et al. [27]	Pertuzumab + trastuzumab/pertuzumab emtansine + Gemcitabine > Cisplatin + gemcitabine in BTC
Majid et al. [28]	Afatinib > erlotinib in PDAC
Assenat et al. [29]	FOLFIRINOX > Trastuzumab + erlotinib + Gemcitabine > Nab-paclitaxel + gemcitabine in PDAC
Tanzeel et al. [30]	Afatinib + IGF-IR/afatinib + dasatinib/dasatinib + gemcitabine > chemotherapy + gemcitabine in PDAC
Rabia et al. [31]	Cetuximab + trastuzumab + pertuzumab > chemotherapy + gemcitabine in PDAC
Marinovic et al. [32]	Cetuximab + trastuzumab > Erlotinib + trastuzumab in PNET
Akihiro et al. [33]	Trastuzumab deruxtecan > FOLFOX/FOLFIRI in BTC
de Vries et al. [34]	Trastuzumab deruxtecan/trastuzumab duocarmazine + chemotherapy > chemotherapy alone in BTC

BTC, biliary tract cancer; PDAC, pancreatic ductal adenocarcinoma; PNET, pancreatic neuroendocrine tumors; >, better treatment outcome in terms of efficacy.

**Table 4:** Author, HER2+ expression and histopathological type, and drug agent.

Author	HER2 Overexpression According to Histopathological Type	Target Agents
Bittoni et al. [23]	1.1% in PDAC	Trastuzumab
Nicola et al. [24]	5–76% overexpression and 1–8% amplification in BTC 0–50% overexpression and 2–29% amplification in PDAC	Trastuzumab, Pertuzumab, Lapatinib, Neratinib, Afatinib
Xiaoping et al. [25]	2.1–23.8% overexpression and 16% amplification in PDAC	Trastuzumab
Elebro et al. [26]	11% in PDAC 7% in BTC 15% in AVC	Erlotinib
Salvatore et al. [27]	19.9% in PDAC 17.4% in BTC 27.9% in AVC	Trastuzumab, Pertuzumab
Majid et al. [28]	16% in resectable PDAC 26% in metastatic PDAC	Dacomitinib
Assenat et al. [29]	58.3% in PDAC	Trastuzumab, Erlotinib
Tanzeel et al. [30]	26.27% in PDAC	Dinaciclib, Afatinib, IGF-IR
Rabia et al. [31]	24% in PDAC with 11% positive for combination of EGFR + *HER2* + HER3	Trastuzumab, Lapatinib, U3-1287
Marinovic et al. [32]	37.9% in nonfunctional PNET 36.11% in functional PNET 42.31% in insulinomas	Trastuzumab, Cetuximab
Akihiro et al. [33]	10–20% in BTC	Trastuzumab Deruxtecan
de Vries et al. [34]	6.3% in PDAC 7.9% in BTC	Trastuzumab Duocarmazine

BTC, biliary tract cancer; PDAC, pancreatic ductal adenocarcinoma; AVC, ampula of Vater cancer; U3-1287, anti-HER3 antibody; PNET, pancreatic neuroendocrine tumors; IGF-IR, insulin-like growth factor 1 receptor inhibitor.

## Data Availability

Data are contained within the article.

## Supplementary Materials

The following supporting information can be downloaded at: https://www.mdpi.com/article/10.3390/jpm14050463/s1, File S1: All Chemo- and Immunotherapics.
