# Supplementary material for: HER2 Overexpression in Periampullary Tumors According to Anatomical and Histological Classification—A Systematic Review
Source: J Pers Med. 2024 Apr 27;14(5):463. doi: 10.3390/jpm14050463 (PMC11122564; doi:10.3390/jpm14050463)
Supplement: Supplementary file 1 [file jpm-14-00463-s001.zip › jpm-2971297-supplementary.pdf]

All Chemo- and Immunotherapics:

Trastuzumab deruxtecan - is an antibody-drug conjugate consisting of the humanized monoclonal antibody trastuzumab covalently linked to the topoisomerase I inhibitor deruxtecan. It is licensed for the treatment of breast cancer or gastric or gastroesophageal adenocarcinoma, and now is in process of testing in pancreatic cancer cells.

FOLFOX - is a chemotherapy regimen for treatment in particular colorectal cancer, made up of the drugs: folinic acid (leucovorin, FOL), fluorouracil (5-FU, F), and oxaliplatin (Eloxatin, OX)

FOLFIRINOX is a chemotherapy regimen for treatment of advanced pancreatic cancer. It is made up of the following four drugs:

FOL – folinic acid (leucovorin), a vitamin B derivative that enhances the effects of 5-fluorouracil (5-FU)

F – fluorouracil (5-FU), a pyrimidine analog and antimetabolite which incorporates into the DNA molecule and stops DNA synthesis;

IRIN – irinotecan (Camptosar), a topoisomerase inhibitor, which prevents DNA from uncoiling and duplicating; and

OX – oxaliplatin (Eloxatin), a platinum-based antineoplastic agent, which inhibits DNA repair and/or DNA synthesis.

Trastuzumab emtansine is an antibody-drug conjugate consisting of the humanized monoclonal antibody trastuzumab (Herceptin) covalently linked to the cytotoxic agent DM1. Trastuzumab alone stops growth of cancer cells by binding to the HER2 receptor, whereas trastuzumab emtansine undergoes receptor-mediated internalization into cells, is catabolized in lysosomes where DM1-containing catabolites are released and subsequently bind tubulin to cause mitotic arrest and cell death.

Gemcitabine is a cytidine analog with two fluorine atoms replacing the hydroxyl on the ribose. As a prodrug, gemcitabine is transformed into its active metabolites that work by replacing the building blocks of nucleic acids during DNA elongation, arresting tumor growth, and promoting apoptosis of malignant cells.

Pertuzumab is a monoclonal antibody used in combination with trastuzumab and docetaxel for the treatment of metastatic HER2-positive breast cancer and targets the extracellular dimerization domain (subdomain II) of HER2, thereby inhibiting ligand-initiated intracellular signaling via the MAP kinase and PI3K pathways. Inhibition of these pathways results in inhibition of cell growth and the initiation of apoptosis, respectively.

Cisplatin /cisplatinum or cis-diamminedichloroplatinum(II) (CDDP) is a platinum-based chemotherapy drug called an alkylating agent. It contains the metal platinum. It damages the DNA of dividing cells in a way that cannot be repaired. This stops or slows the growth of cancer cells and other rapidly dividing cells and causes them to die.

Trastuzumab duocarmazine (SYD985) was obtained by conjuring trastuzumab to duocarmycin via a cleavable linker. Isolated from Streptomyces in the late 1970s, duocarmycin is a DNA alkylating agent consisting of the DNA alkylating part and the DNA binding part that binds irreversibly to DNA spiral

grooves through covalent bonds, blocking DNA replication and transcription, destroying nucleic acid structure, and eventually leading to cell death.

Erlotinib is a tyrosine kinase inhibitor (TKI) which is a type of cancer growth blocker. It blocks proteins on cancer cells that encourage the cancer to grow. These proteins are called epidermal growth factor receptors (EGFR). Erlotinib binds to the epidermal growth factor receptor (EGFR) tyrosine kinase in a reversible fashion at the adenosine triphosphate (ATP) binding site of the receptor.

Nab paclitaxel (Protein-bound paclitaxel), also known as nanoparticle albumin–bound paclitaxel or nab-paclitaxel, is a novel formulation of paclitaxel that does not require solvents such as polyoxyethylated castor oil and ethanol. Use of these solvents has been associated with toxic response, including hypersensitivity reactions and prolonged sensory neuropathy, as well as a negative impact in relation to the therapeutic index of paclitaxel. nab-paclitaxel displays greater antitumor activity and less toxicity than solvent-base paclitaxel.

Cetuximab is a recombinant chimeric human/mouse IgG1 monoclonal antibody that competitively binds to epidermal growth factor receptor (EGFR) and competitively inhibits the binding of epidermal growth factor (EGF). In vitro, cetuximab was shown to mediate anti-tumor effects in numerous cancer cell lines and human tumor xenografts.

Afatinib is a 4-anilinoquinazoline tyrosine kinase inhibitor in the form of a dimaleate salt. Afatinib irreversibly inhibits human epidermal growth factor receptor 2 (Her2) and epidermal growth factor receptor (EGFR) kinases. Afatinib is not only active against EGFR mutations targeted by first generation tyrosine-kinase inhibitors (TKIs) like erlotinib or gefitinib, but also against less common mutations which are resistant to these drugs.

Dasatinib is an orally available multikinase inhibitor which emerged as a potent inhibitor of imatinib-resistant protein tyrosine kinase (KIT) activation loop mutants and it is able to induce apoptosis in mast cell and leukemic cell lines expressing these mutations.

Capecitabine is a prodrug, that is enzymatically converted to fluorouracil (antimetabolite) in the tumor, where it inhibits DNA synthesis and slows growth of tumor tissue.
